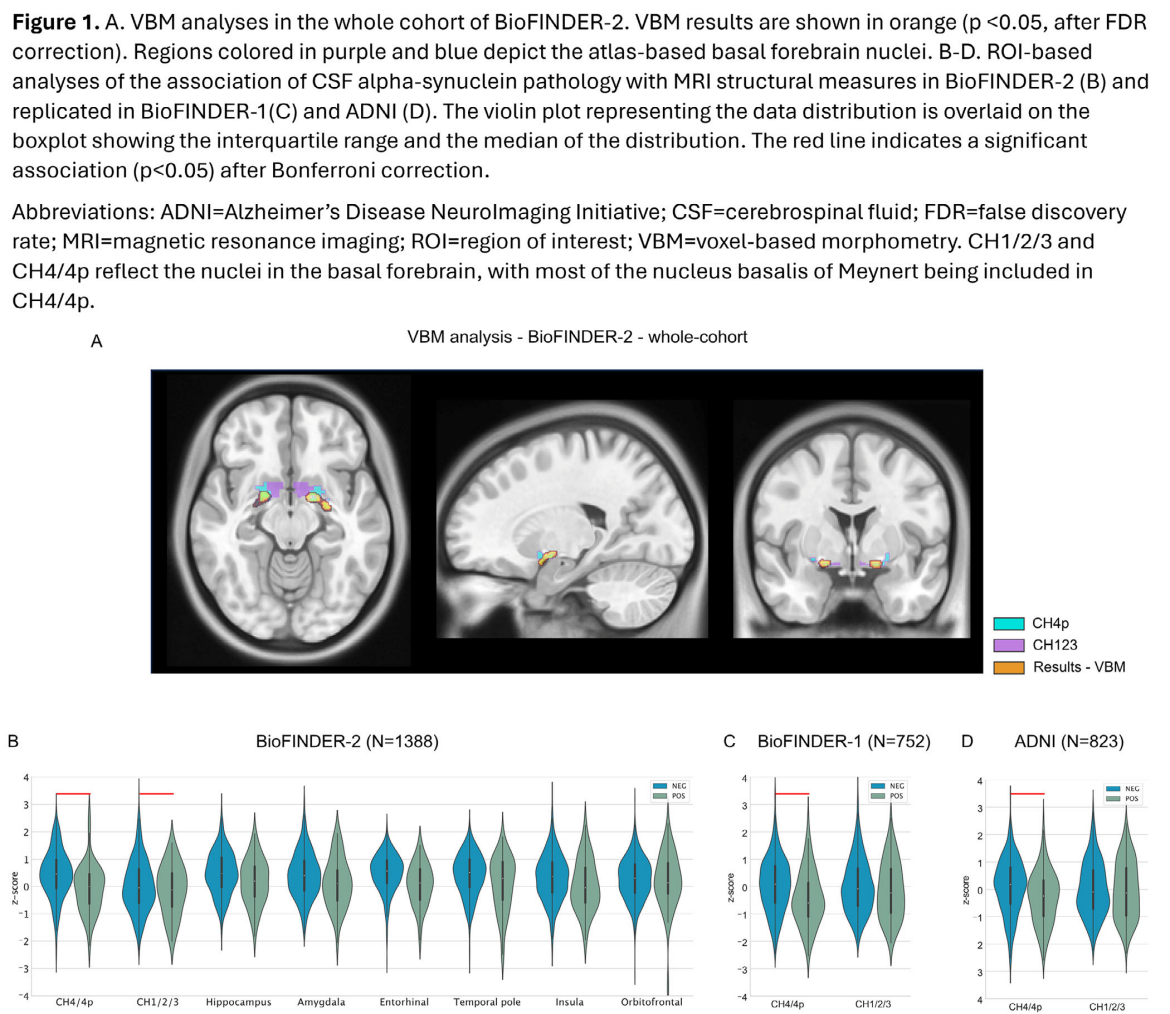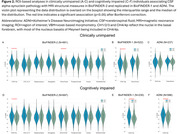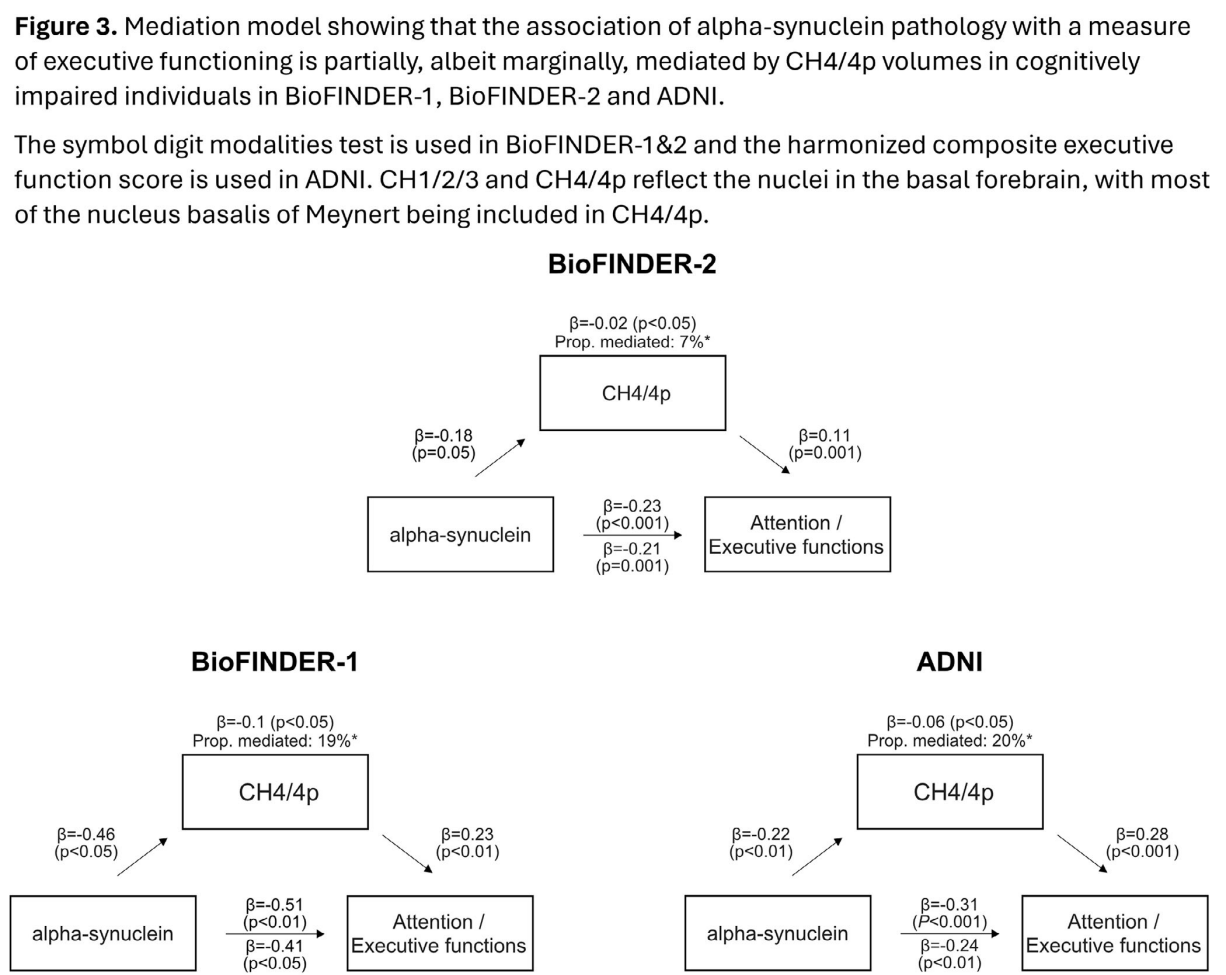# The MRI signature of alpha‐synuclein pathology in preclinical stages and in a memory clinic population

**DOI:** 10.1002/alz.088839

**Published:** 2025-01-09

**Authors:** Laura E.M. Wisse, Nicola Spotorno, Olof Strandberg, Danielle van Westen, Niklas Mattsson‐Carlgren, Sebastian Palmqvist, Erik Stomrud, Piero Parchi, Oskar Hansson

**Affiliations:** ^1^ Department of Clinical Sciences Lund, Lund University, Lund, Lund Sweden; ^2^ Lund University, Lund Sweden; ^3^ Clinical Memory Research Unit, Department of Clinical Sciences Malmö, Faculty of Medicine, Lund University, Lund Sweden; ^4^ Diagnostic Radiology, Department of Clinical Sciences, Lund University, Lund Sweden; ^5^ Clinical Memory Research Unit, Lund University, Malmö Sweden; ^6^ Clinical Memory Research Unit, Lund University, Lund Sweden; ^7^ IRCCS Istituto delle Scienze Neurologiche di Bologna, Bologna Italy; ^8^ Clinical Memory Research Unit, Department of Clinical Sciences, Lund University, Lund Sweden

## Abstract

**Background:**

Alpha‐synuclein pathology underlies Lewy body diseases and can also occur comorbid to other neurodegenerative pathologies. The lack of an in vivo measure for alpha‐synuclein pathology until recently has limited thorough characterization of its brain atrophy pattern, especially during early disease stages. We therefore aimed to assess the association of alpha‐synuclein pathology in cerebrospinal fluid (CSF) with magnetic resonance image (MRI) structural measures in three independent cohorts, and separately in clinically unimpaired (CU) and cognitively impaired (CI) individuals, the latter reflecting a memory clinic population.

**Method:**

Individuals from BioFINDER‐1 (N=752), BioFINDER‐2 (N=1388) and the Alzheimer’s Disease Neuroimaging Initiative (ADNI; N=823) with CSF alpha‐synuclein seed amplification assay results were included. T1‐weighted MRIs were processed with voxel‐based morphometry (VBM) and for region of interest (ROI) analyses with FreeSurfer 6.0 and an automated pipeline for basal forebrain nuclei CH1/2/3 and CH4/4p (=nucleus basalis of Meynert). All analyses were corrected for age, sex, intracranial volume (volumetric measures), CSF Aβ42/40 (ADNI: Aβ42), phospho‐tau181 and cognitive status (only whole cohort). Executive functioning was assessed with the symbol digit modalities test (BioFINDER‐1&2) or the harmonized composite executive function score (ADNI).

**Result:**

In BioFINDER‐2, VBM analyses in the whole cohort revealed a specific association between alpha‐synuclein pathology and the cholinergic CH4/4p nuclei (Figure 1). Next, ROI‐based analyses, focused on regions involved in the cholinergic system, revealed that alpha‐synuclein pathology was indeed associated with smaller CH4/4p volumes, and with smaller CH1/2/3 volumes in BioFINDER‐2. Even in CU individuals, alpha‐synuclein pathology was associated with smaller CH4/4p volumes and entorhinal cortex thickness; only the former association was found in CI individuals (Figure 2). Overall, the association between alpha‐synuclein pathology and CH4/4p volume was replicated in BioFINDER‐1 and ADNI. In CI individuals, CH4/4p volumes partially mediated the association of alpha‐synuclein pathology with executive functioning in all cohorts (Figure 3).

**Conclusion:**

The findings suggest that atrophy in relation to alpha‐synuclein pathology is restricted and clearly affects the nucleus basalis of Meynert already during preclinical stages. Further, in memory clinic populations with cognitive impairment, alpha‐synuclein pathology is also associated with nucleus basalis of Meynert atrophy, which seems to partially mediate alpha‐synuclein‐induced executive impairment.